# Unravelling molecular mechanisms from floral initiation to lipid biosynthesis in a promising biofuel tree species, *Pongamia pinnata* using transcriptome analysis

**DOI:** 10.1038/srep34315

**Published:** 2016-09-28

**Authors:** Rachapudi V. Sreeharsha, Shalini Mudalkar, Kambam T. Singha, Attipalli R. Reddy

**Affiliations:** 1 Department of Plant Sciences, University of Hyderabad, Hyderabad, 500046, India

## Abstract

*Pongamia pinnata* (L.) (Fabaceae) is a promising biofuel tree species which is underexploited in the areas of both fundamental and applied research, due to the lack of information either on transcriptome or genomic data. To investigate the possible metabolic pathways, we performed whole transcriptome analysis of Pongamia through Illumina NextSeq platform and generated 2.8 GB of paired end sequence reads. The *de novo* assembly of raw reads generated 40,000 contigs and 35,000 transcripts, representing leaf, flower and seed unigenes. Spatial and temporal expression profiles of photoperiod and floral homeotic genes in Pongamia, identified GIGANTEA (GI) - CONSTANS (CO) - FLOWERING LOCUS T (FT) as active signal cascade for floral initiation. Four prominent stages of seed development were selected in a high yielding Pongamia accession (TOIL 1) to follow the temporal expression patterns of key fatty acid biosynthetic genes involved in lipid biosynthesis and accumulation. Our results provide insights into an array of molecular events from flowering to seed maturity in Pongamia which will provide substantial basis for modulation of fatty acid composition and enhancing oil yields which should serve as a potential feedstock for biofuel production.

Decreasing the fossil fuel consumption and reconciling the worsening global environmental conditions are fundamental concerns of the society in this industrial era. The development and use of alternative fuels, including bioethanol and biodiesel are predicted to significantly alleviate the problems caused by the usage of fossil fuels. An imminent biofuel tree *Pongamia pinnata* (L.) (Family: Fabaceae), is a native species of India which can be grown in diverse tropical and subtropical marginal lands of the world. It is a drought and salinity tolerant, semi-deciduous, nitrogen fixing tree which grows up to 15–20 meters in height with a large canopy^1^. The oil content of Pongamia seeds ranges from 35 to 40% of seed dry weight and 55% of it is oleic acid which is the ideal fatty acid for good quality biodiesel production. Upon trans-esterification, the Pongamia oil, with a blend of diesel, can be applied in automobiles without any further modification of engines. Pongamia has a long life cycle, usually sets flowering after 4–5 years of plantation and takes 9–11 months to form a mature pod after anthesis. There are many positive attributes that could be potentially achieved by understanding the genetics and genomics of Pongamia. For instance, the oil content of Pongamia which is around 35% could be increased to about 50% with higher oleic acid content and also the fruit maturity time could be reduced. But, limited genetic resources and long production cycles have constrained the molecular breeding programs aimed at better oil quality and seed yields in this potential biofuel tree species.

Flowering time, fertilization and seed development are inter-related and determine the yield of certain promising biofuel tree species, including Pongamia. Understanding the molecular mechanisms that control the onset of reproductive events and development of the seeds is crucial for improving the biofuel feedstock. Photoperiod and vernalization pathways have been reported as major control mechanisms to synchronize environmental cues with the internal rhythm^2^,^3^,^4^. The former promotes flowering in response to increasing day length and the latter enables induction of flowering following a prolonged exposure to cold. In order to trigger flower initiation at the precise time and in the right conditions, circadian clocks perceive and integrate both environmental and endogenous signals, which in turn activate mobile florigen and other related proteins to induce flowering. Besides the floral promotion pathways, mutation studies in *Arabidopsis* have also revealed the existence of genes that repress the floral transition^5^. Exploiting these genes through genetic engineering by rendering knowledge obtained from the model plants like *Arabidopsis* and sequence information obtained from transcriptome will have substantial impact on floral transitions in Pongamia. However, lack of publicly available sequence information is a hindrance to the efforts for improving seed oil quality and floral transition period in non-model and non-edible biofuel crops like Pongamia.

In many seeds, triacylglycerols (TAGs) that accumulate during the maturation phase of embryo and/or endosperm act as major storage reserves of carbon and in the due course of germination, they support the establishment of the seedling^6^. Fatty acid (FA) metabolism and the enzymes involved in it play an important role in plant morphology, growth, seed development and stress responses. Earlier reports have shown that FATB mutant of *Arabidopsis* resulted in reduced concentrations of palmitate and stearate and affect both plant growth and seed development^7^. Similarly, enoyl-CoA reductase and stearoyl – ACP desaturase had essential roles in endocytic membrane trafficking and defence responses respectively by regulating the oleic acid contents^8^,^9^. Also, owing to the commercial importance of plant lipids, several attempts have been made to alter seed TAG composition through genetic engineering. For instance, *Arabidopsis* and *Camelina* transgenic plants were engineered to produce oil with high omega-3/omega-6 ratio and high DHA^10^. Engineering of key FA biosynthetic enzymes, including fatty acid desaturase 2 (FAD2) and fatty acid elongase 1 (FAE1) has resulted in high oleic acid containing lines of *Camelina* and soybean^11^,^12^. Oil accumulation depends on the seed development patterns which show great diversity in duration of maturity and oil formation stages. Knowledge about temporal expression patterns of oil biosynthetic enzymes is crucial to understand reprogramming strategies of oil biosynthesis as well as modification in fatty acid composition.

High throughput deep sequencing of transcriptome is a promising and powerful tool to identify the key genes associated with species-specific exotic FA biosynthetic enzymes and molecular marker development. Of late, several non-model organisms, whose reference genome sequence was absent, were sequenced and annotated using platforms such as Roche/454, AB SOLiD and Illumina^13^,^14^,^15^,^16^,^17^. Recently, salt responsive genes were identified upon transcriptome sequencing of leaf and root tissues of *Pongamia pinnata* using Illumina platform^18^. In the same year, another group had claimed the chloroplastic and mitochondrial genome of Pongamia through second generation DNA sequencing^19^. Further, Pavithra *et al.*^20^, reported the FA profiles of seeds at different developmental stages of Pongamia. Very recently, parallel to the current study, Huang *et al.*^21^, reported the seed transcriptome of Pongamia which provided valuable information about lipid biosynthetic genes and SSR markers. But the whole transcriptome information, together with gene expression patterns of lipid biosynthetic enzymes during seed development and flowering pathway genes, are far from being characterised in Pongamia. In the present study, we constructed the paired end cDNA library from pooled RNA isolated from leaf, flower, pod and seed tissues of mature Pongamia tree and sequenced using Illumina TrueSeq protocol on Illumina NextSeq 500 platform. We also data mined circadian clock genes and lipid biosynthetic genes in Pongamia and reported their homology with other model organisms. Our data provide a comprehensive information on Pongamia transcriptome and temporal expression of candidate genes involved in lipid accumulation and flowering which can be applied to molecular breeding programs for improving the seed oil content in Pongamia.

## Results

### Sequencing and *de novo* assembly of Pongamia transcriptome

We generated a total of 24 004 632 paired end sequence reads, each 76 bp in length, encompassing about 2.8 GB of sequence data in fastQ format. After stringent filtering of sequence data for low-quality reads and reads containing primer/adaptor sequences, we obtained a total of 22 158 278 high quality sequence reads (with phred quality score of <20). The final data set comprising ~22 million very high-quality reads were used for optimization of *de novo* assembly and analysis of Pongamia transcriptome. All processed reads were assembled into contigs without any reference (*de-novo*) using velvet – 1.2.10 software. Assembly was tried on various hash lengths (k-mers) and 41 was selected as the best hash-length. Best k-mer is decided on various parameters including: number of contigs, total number of reads used, total contig length and number of non-ATGC characters. A total of 42,724 contigs were generated with maximum and minimum lengths of 11,665 and 200 bp respectively with an average read length of 639.7 bp. Contigs were then processed into transcripts (spliced isoforms) using oases – 0.2.8 software. The majority of high quality reads (81.77%) were assembled to generate a total of 36,047 transcripts that ranged from 200–30, 656 bp length with an average length of 937.586 bp. The number of transcripts were decreased as the length increased with maximum number of transcripts falling in the range of 0–100 bp followed by 100–500 bp. The N50, N90 and rpkm values of Pongamia transcriptome along with other related parameters were presented in Supplementary Table S1. The average GC content of Pongamia transcripts was 48% and has a higher proportion of transcripts in the range of 40–45%, followed by 45–50%, resulting much broader GC content range (Supplementary Fig. S1).

### Functional annotation and characterisation of Pongamia transcripts

**Figure 1:**
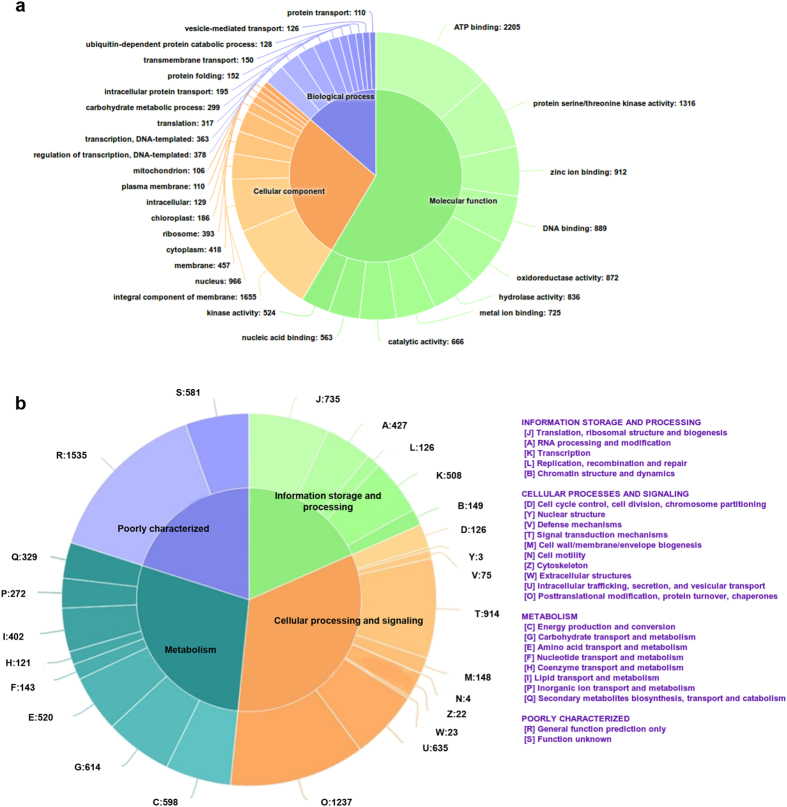
GO and KOG classification. (**a**) Gene ontology distribution of the transcripts into biological process, molecular function and cellular component. The number of transcripts encoded for each category is represented. (**b**) Comparison of transcripts with the KOG database and classification into groups such as metabolism, information storage and processing, cellular processes and signalling resulting in 25 different categories.

The assembled transcripts were annotated against NCBI-BLAST 2.2.29 using GeneMark software. A total of 47,461 genes/proteins were predicted of which 25,112 proteins were annotated with either Swiss-Prot (108) or TrEmbL (25, 004) databases. Most of the predicted proteins in Pongamia showed homology in UniProt with *G. max* (44%) and *G. soja* (22%) followed by *P. vulgaris* (21%) and other Papilinoideae members (Supplementary Data 1). The transcripts that show significant homology to the genes against UniProt database were selected for GO annotation. A total of 16,146 (45%) transcripts were assigned with at least one GO term, in which 9508 (26.37%) were assigned in biological process category, 4420 (12.26%) were assigned in cellular component category and 2218 (6.15%) were assigned to molecular function category (Fig. 1a). Among the various biological processes, ignoring unknown and other biological process categories, ATP binding (2205) and protein serine/threonine kinase activity (1316) were highly represented. The genes involved in other biological processes such as zinc ion binding, DNA binding, oxidoreductase activity, hydrolase activity and metal ion binding and those having catalytic activity were also identified through GO annotations (Fig. 1a). Similarly, genes involved in transcription and transcription regulation were mostly represented in molecular function category, followed by carbohydrate, protein metabolism and transport (Fig. 1a). Integral membrane components and nucleus were most represented among the cellular components followed by membrane and cytoplasm. Also, we annotated 13,764 Pongamia genes to KOG (Eukaryotic Orthologous Groups) database which aids in identification and phyletic classification of the orthologous proteins, coded in whole genome of almost 21 organisms including bacteria, algae and eukaryotes^22^. The resulting KOG annotation grouped the transcripts into three functional categories: cellular processing and signalling (3214; 31%); metabolism (2999; 30%) and information storage and processing (1945; 19%) and rest of the genes resulted in poorly characterized annotations (Fig. 1b) (Supplementary Data 2). We identified a total of 4148 SSRs in which mono-nucleotide SSRs represented the largest fraction (36.4%) followed by tri-nucleotide (31.3%) and di-nucleotide (28.8%) SSRs (Supplementary Fig. S2). Pongamia transcripts also contained a quite significant number of tetra- (79), penta- (31) and hexa – nucleotide (28) SSRs though their representation is small in total SSR pool.

### Sequence similarity of Pongamia transcripts with other plants

**Figure 2:**
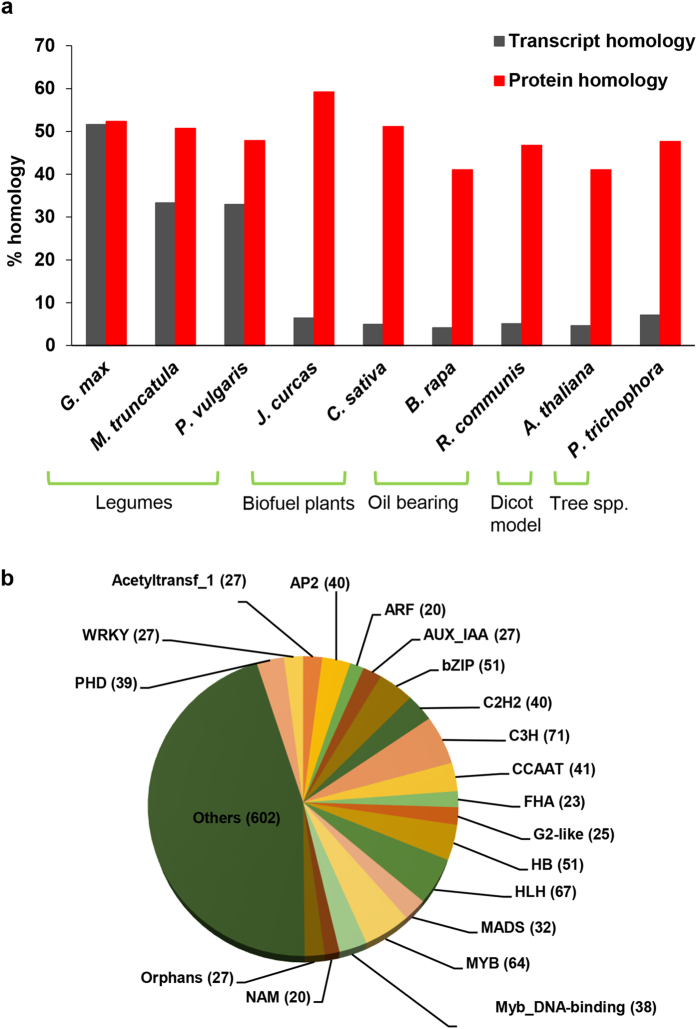
(**a**) Sequence similarity and percentage homology of Pongamia transcripts and putative proteins with other related organisms. (**b**) Classification of transcripts into transcription factor families. Number of genes represented in each family were indicated in parenthesis.

The transcripts of Pongamia were analysed for similarity against the unigene datasets of legume crops, biofuel plants and other oil bearing plants belonging to different families using TBLASTX search. An E-value cut-off threshold of 1E – 05 was considered to define a significant hit. The largest number of Pongamia transcripts showed significant similarity with soybean putative mRNA sequences (53%) followed by *M. truncatula* (35%). While, Pongamia showed little conservation with oil bearing trees of other families (Fig. 2a). We also analysed the sequence conservation of translated Pongamia transcripts with proteomes of selected plant species. Putative Pongamia proteins showed maximum homology with the biofuel plant *J. curcas* (59.2%) followed by *C. sativa* (52%) and *G. max* (53%) (Fig. 2a). Our analysis also showed that 53% of Pongamia transcripts are having homology with legumes indicating these genes are legume specific (Supplementary Data 3).

### Identification of transcription factor families

We identified the transcription factor encoding transcripts by sequence comparison to known transcription factor gene families in Plant TFDB (Transcription factor data base). In total, 1332 putative transcription factor genes distributed in at least 18 families were identified representing 3.7% of Pongamia transcripts (Supplementary Data 4). Genes encoding for C3H, HLH, MYB, bZIP and HB transcription factor families were abundantly expressed while minimum number of transcripts were observed for NAM, ARF, FHA, MADS and WRKY families (Fig. 2b). Further, we annotated and analysed transcription factors exclusive to lipid biosynthetic pathway which resulted in identification of genes belonging to MYB, PLATZ, GRAS, MYB-related, bHLH8, CCAAT, G2-like and PHD transcription factor families.

### Pathway mapping of transcripts by KEGG

Ortholog assignment and mapping of the contigs to the biological pathways were performed using KEGG automatic annotation server (KAAS). All the contigs were compared against the KEGG database using BLASTX with threshold bi-score value of 60 (default). It assigned Enzyme Commission numbers for 2784 contigs, and they were mapped to respective pathways (Supplementary Data 5). Among the mapped contigs, 1709 were identified as genes involved in metabolic pathways of major biomolecules such as carbohydrates (306, 11%), amino acids (252, 9%), lipids (177, 6.3%), nucleotides (150, 5.3%), cofactors, vitamins (130, 4.6%), glycans (71, 2.5%), terpenoids (74, 2.5%). The KEGG pathway analysis also showed that 186 and 69 contigs represent the energy and secondary metabolites metabolisms respectively. A total of 247 transcripts that represent enzymes involved in carbon metabolism, fatty acid metabolism, degradation of aromatic compounds, biosynthesis of amino acids as well as 2-oxocarboxylic acid metabolism were also identified. Further, the mapped contigs also represented the genes involved in genetic information processing that include, translation (12.3%), folding, sorting and degradation (9.5%), transcription (5.7%) as well as replication and repair (4.5%). Cellular processes (transport and catabolism, cell motility, cell growth and death, cell communication) and environmental information processing (membrane transport, signal transduction, signalling molecules and interaction) are other minor groups represented in the KEGG annotation of Pongamia. The KAAS analysis also represented genes involved in biosynthesis of karanjin, ansamycin and siderophore thus substantiating the insecticidal, medicinal and anti-bacterial properties of Pongamia respectively (Supplementary Data 5). Further, we studied genes involved in the following major metabolic events which had a prominent role in improving the yield and oil quality related traits in Pongamia.

### Genes involved in circadian rhythms

**Figure 3:**
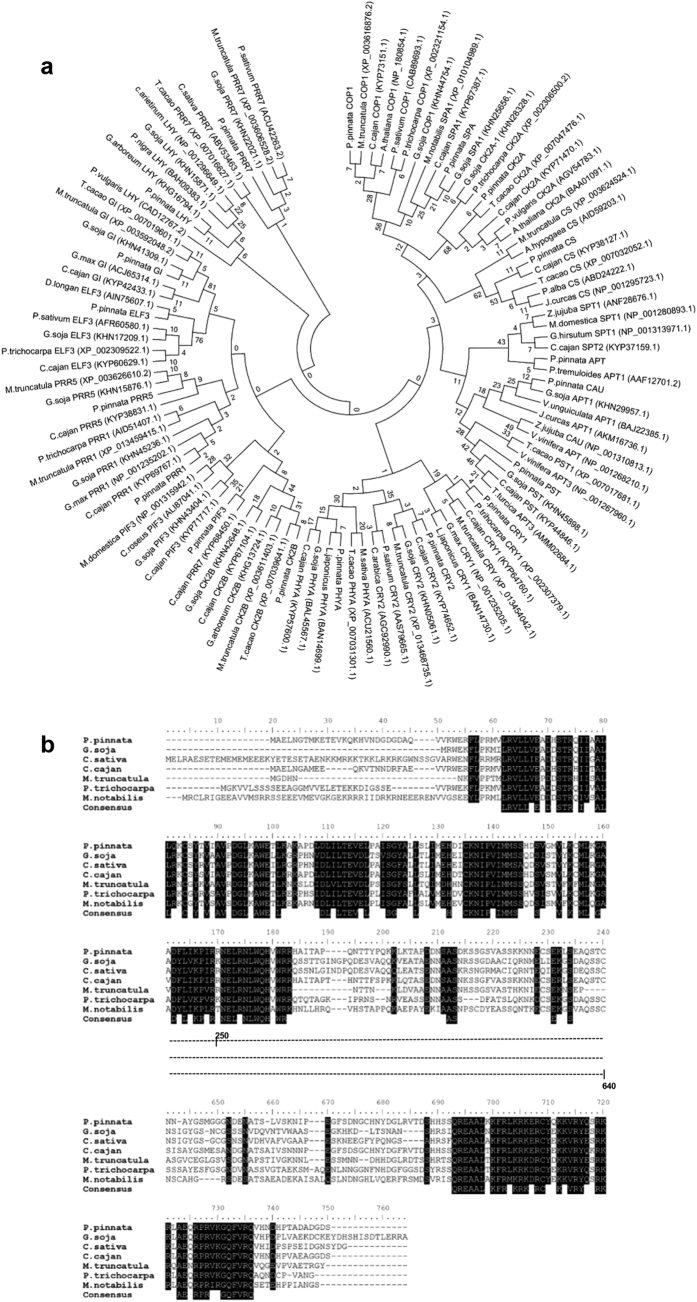
Sequence homology and phylogenetic analysis. (**a**) Pongamia flowering related genes and their phylogenetic relationship with ortholog proteins from other related organisms. The evolutionary history was inferred using the Neighbor-Joining method. The bootstrap consensus tree inferred from 1000 replicates is taken to represent the evolutionary history of the taxa analyzed. Branches corresponding to partitions reproduced in less than 50% bootstrap replicates are collapsed. The evolutionary distances were computed using the JTT matrix-based method and are in the units of the number of amino acid substitutions per site. All positions containing gaps and missing data were eliminated. Evolutionary analyses were conducted in MEGA7. The accession numbers of all the genes were given in parenthesis. (**b**) multiple sequence alignment of the PpPRR5 with orthologs from other related organisms depicting the conserved C - terminal and N - terminal regions (highlighted black). BioEdit software was used to align the sequences.

Our transcriptome data represented 25 key genes including TFs that are involved in the regulation of floral meristem identity, photoperiod as well as vernalization pathways (Table 1). Majority of the genes were having sequence homology with *G. max* followed by *M. truncatula* which shows the evolutionarily conserved relationship between legumes. The phylogenetic relationship of Pongamia flowering genes with other related organisms was deduced (Fig. 3a). ORF sequence analysis unfold the complete protein coding sequence of all the genes and the polypeptide information was presented (Table 1). Genes like Flowering locus T (FT), GIGANTEA (GI), Chalcone synthase (CS), PRR1/TOC1, PRR5, PRR7 represented in this study were known to promote flowering through photoperiod regulation under long day conditions. The Pseudo-receiver (PR) domain at the N-terminal region and CCT domain at the C-terminal region which are characteristic conserved domains of PRR protein family were identified in Pongamia compared with other related organisms (Fig. 3b). Other genes like PST, SPT, APT, CAU, AGA which encode TFs play a key role in defining floral meristem identity. The transcriptome also represented COP1, SPA1, PHYA, PHYB and CK2A genes which inhibit flowering process by repressing CONSTANS (CO) gene during prolonged cold periods through vernalization responses.

### Spatial and temporal profiling revealed the diurnal nature of clock genes

**Figure 4:**
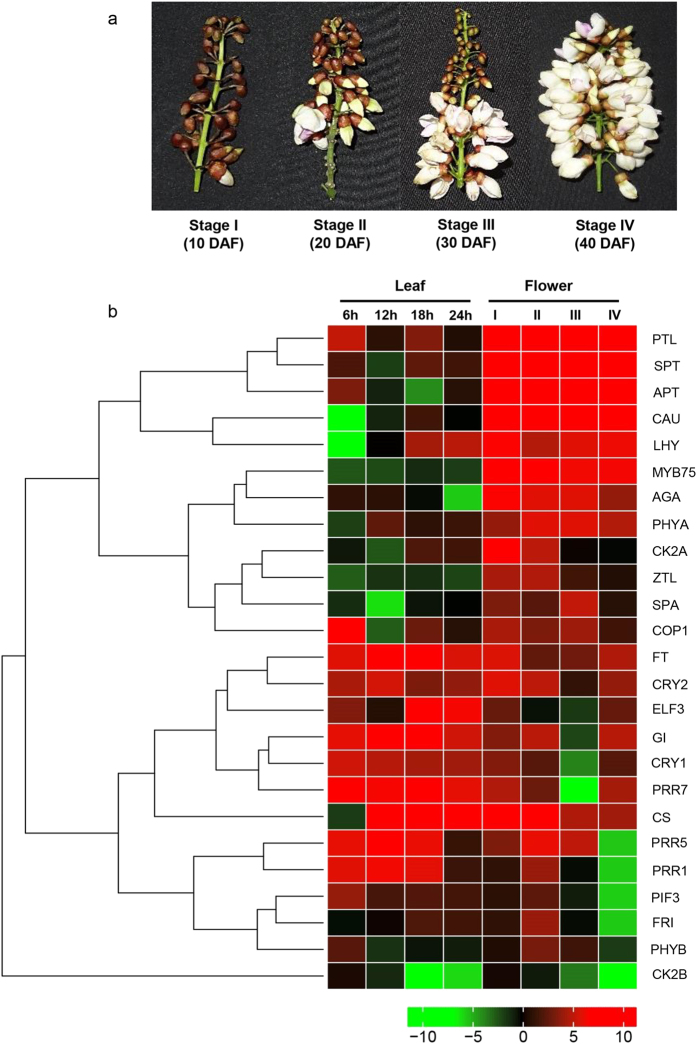
(**a**) Infloresence of Pongamia at four different developmental stages used in gene quantification studies. (**b**) Heatmap representing expression profiles of flowering related genes and transcription factors in Pongamia leaves and flowers. Heatmap was constructed using gplot in R-package. For gene abbreviations see Table 1.

To understand the diurnal behaviour of circadian clock genes and expression trends of floral homeotic genes, we quantified the expression of key flowering pathway genes in leaf tissues collected at four different time points in a day (6, 12, 18, 24 hrs) as well as in inflorescence of four different stages (10, 20, 30, 40 days after flowering - DAF) (Stage 1, 2, 3 and 4 respectively) in a field grown Pongamia plant (Fig. 4a). We selected photoperiod pathway genes, vernalisation genes and certain crucial transcription factors which act as floral homeotic proteins and a heatmap was constructed based on their expressin profiles (Fig. 4b). The genes were divided into three clusters based on their expression profiles (i) circadian clock genes that function in initiation of flowering through photoperiod pathway: genes like PRR1, PRR5, PRR7 showed time dependent regulation in leaves wherein, the three genes showed significant up regulation in the morning and decreased expression during dusk (Fig. 4b). Contrastingly, ELF3 which is an evening gene showed peak expression during 18–24 hrs. GI which acts upstream to FT, is a major component of leaf generated mobile florigen, showed coordinated expression with FT wherein, peak expression at 6 and 12 h of day was observed. At the same time, they showed minimal expression in inflorescence at all stages. (ii) Circadian clock genes that repress flowering process: COP1-SPA1 complex which operates in dark conditions to degrade CO protein by polyubiquitination showed downregulation as the day light progressed. PHYA, PHYB and CK2A were only expressed during midday in leaves but showed significant up regulation in earlier stages of inflorescence (Fig. 4b). (iii) TFs which operate in the process of flower development and photoperiod: APT, PTL, AGA, CAU and SPT showed significant and constitutive expression in all stages of inflorescence. The expression of PTL, SPT and APT was significantly peaked at stage 2 of inflorescence whereas, CAU, AGA showed higher expression at stage 1. MYB75 TF expression was significantly high in flowers and roots compared to leaf tissue. LHY which is a MYB – related TF that binds to the promoter region of PRR1/TOC1 thereby repressing their expression to postpone the flowering process, showed basal level of expression in leaves and significant up regulation in flowers (Fig. 4b).

### Genes involved in membrane and storage lipid metabolism

**Figure 5:**
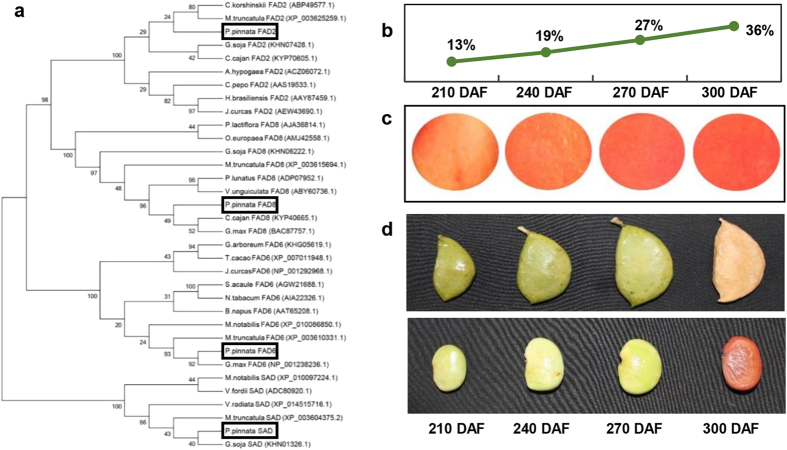
(**a**) Phylogenetic analysis: PpFAD2, PpFAD6, PpFAD8, PpSAD and their phylogenetic relationship with ortholog proteins from other related organisms. (**b**) Percentage of oil content during four developmental stages of Pongamia seed. (**c**) oil-red staining of seed endosperm showing oil accumulation patterns in Pongamia seed. (**d**) four developmental stages of Pongamia seed used in quantification studies of lipid biosynthetic genes.

A total of 203 transcripts corresponding to 136 unigenes were identified and grouped into 14 categories of various lipid metabolic pathways (Supplementary Table S3). The KEGG annotation, sorted out 264 unigenes representing all essential enzymes involved in fatty acid biosynthesis, elongation, degradation as well as glycerolipid and phospholipid metabolisms. From our data, it is evident that 41 genes have mapped to transcripts at two different loci indicating the diploid nature in Pongamia and 20 genes have duplicate contigs at the same locus presuming the gene duplication and the presence of paralogs. The overall lipid metabolism is an interplay between carbohydrate and fatty acid stoichiometric profiles and can be viewed as three major events: (i) pyruvate to Acetyl CoA synthesis (ii) FA synthesis from Acetyl CoA (iii) TAG assembly and degradation. Our transcriptome data represented all the major rate limiting enzymes involved in these three categories (Table 2). We also analysed the homology of Pongamia transcripts with respective organisms having maximum sequence coverage. ORF analysis indicated that the putative Pongamia transcripts were having full length sequences and the polypeptide information (mass and pI) of translated putative transcripts suggested that the proteins involved in the oil biosynthetic pathway are functional mostly at basic pH (Table 2). We further searched the orthologs for PpFAD8, PpFAD2, PpFAD6 and PpSAD to establish the phylogenetic relationship of Pongamia with other legumes, oil bearing trees and crop plants. Our data demonstrated PpFAD6 and PpSAD are part of a small clades consisting of respective genes from *Glycine max* and *Glycine soja* (Fig. 5a). Intriguingly, PpFAD8 was grouped with neither legumes nor biofuel trees and formed a separate clade indicating its distinct evolutionary conservation (Fig. 5a). Also, phylogeny of certain key flowering related genes of Pongamia was established. Considerable number of transcripts were also found for arachidonic acid (4%) and linoleic acid (2%) metabolism in our transcriptome data. Besides lipids for oil biosynthesis, enzymes involved in membrane lipid biosynthesis, including glycerolipid metabolism, glycerophospholipid metabolism, sphingolipid metabolism, steroid biosynthesis, ether lipid metabolism and cutin as well as suberin and wax synthesis were also identified through our data which corroborated that the transcriptome has covered all the genes involved in lipid biosynthesis signifying the depth of the sequencing (Supplementary Table S3).

### Profiling of lipid biosynthetic genes revealed stage specific FA synthesis during seed development

**Figure 6:**
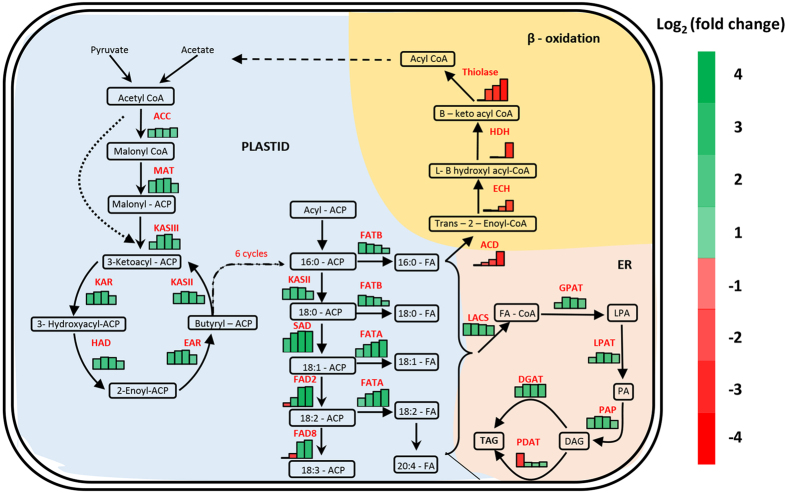
Schematic representation of oil biosynthetic pathway. The relative expression levels of genes involved in fatty acid biosynthesis, elongation, TAG assembly and degradation were analysed through qRT PCR. The fold change was log_2_ transformed and represented as a bar diagram with colour coding against each enzyme. LPA: lysophosphatidic acid; PA: phosphatidic acid; DAG: diacylglycerol; TAG: triacylglycerol. For gene abbreviations see Table 2.

Our data on seed oil accumulation showed that, Pongamia accumulate oil actively after 210 DAF and progressed with the pod development till maturity (300 DAF) (Fig. 5b). The oil red staining of total lipids in the seed sections also showed a gradual increase in the lipid content of seeds during the four stages (Fig. 5c). Further, mRNA expression levels of genes coding for oil biosynthetic enzymes were studied during four stages of seed development which include: mature green pod stages (210 DAF, 240 DAF and 270 DAF) (Stage 1, 2, 3 respectively) and late dark brown pod stage (300 DAF) (stage 4) that were normalised with immature green pod stage (150 DAF) (Fig. 5d). Expression profiles of all the genes were shown in a schematic representation of lipid metabolism (Fig. 6). Based on their expression levels at four different stages, the lipid biosynthetic genes were categorised into 4 groups: (a) those which expressed in a bell shaped manner which include, MAT, KASII, KASIII, KAR, HAD, EAR, LPAT, PAP. These genes showed peak expression during 2^nd^ and 3^rd^ stages and decreased thereafter. (b) Those which showed a gradually decreased expression as the development progressed: FATB and PDAT. (c) Those which showed increased expression towards the development of pod: Thiolase, HDH, ECH, ACD which majorly involved in β-oxidation and (d) those which expressed constantly throughout the pod development like ACC, LACS, DGAT (Fig. 6).

## Discussion

The next generation sequencing technologies and bioinformatics tools enable assembly and annotation of short reads into expressed sequence data, particularly for non-model organisms without a known reference. In this study, using the Illumina NextSeq platform, we characterized whole transcriptome of a non-model legume biofuel tree Pongamia, for which the sequence data are limited so far in the public databases. Genes related to lipid biosynthesis, flowering cycle and flavonoid biosynthesis were emphasised and the transcript information was further used to understand the temporal expression patterns of oil biosynthetic and flowering related genes in Pongamia. The total RNA from the four tissues were pooled, and normalized cDNA was synthesised which remarkably reduces the frequency of abundant transcripts and increases the rate recovery of unique transcripts^23^. Upon sequencing and *de novo* assembly, we selected a total of 92% of sequenced raw reads by stringent filtering to annotate into functional transcripts belonging to crucial metabolic pathways of leaf, flower and seed. The average length of Pongamia unigenes (937.58 bp) was more than those of reported in other related species like chickpea (523 bp), peanut (619 bp), alfalfa (803 bp), as well as in a recent report on Pongamia (787 bp) but shorter than those of *Camelina* (1198 bp)^15^,^24^,^25^,^26^. The GC content (ratio of guanine and cytosine) which ranges from 20 to 72% among different organisms, is an important criterion for establishing the phylogenetic and evolutionary relationships among various species. Our analysis revealed that the average GC content of Pongamia transcripts (48%) was little lower than the *C. sativa* (49%) and higher than *J. curcas* (43%) and in other Pongamia report (44.77%) which explains the complexity and diversity of the transcriptome sequencing^15^,^21^. The parameters like mean length of unigenes, GC content, N50 value of present data show the increased coverage and depth of the sequencing. Annotation of transcripts to UniProt resulted in identification of putative proteins which corresponded to various metabolic pathways. In the current study, we outlined SSR markers identified in Pongamia, which act as important resource in gene mapping and marker assisted molecular breeding. More emphasis on Pongamia EST-SSR markers development, characterization and validation was given in a recent study^21^. Certain transcription factors identified in the current study were reported to play an important role in regulation of gene expression in various metabolic and signalling pathways like fatty acid biosynthesis (MYB, PLATZ), elongation (MYB-related, Bhlh), palmitoleate biosynthesis (MYB), oleate biosynthesis (bHLH, GRAS) stearate biosynthesis (G2-like) and fatty acid degradation (PHD, CCAAT)^15^. Interestingly, MYB and MYB related transcription factors which were deciphered in this study are involved in regulation of circadian rhythms and flowering.

Flowering, which is regulated by circadian rhythms, determines production of seeds and yield of the plant. Circadian clock genes integrate the environmental signals required for flowering and also help in adaptation of plants to different geographical locations^4^. It is of great significance to know the sequence information of genes involved in circadian rhythms and floral transitions to understand and alter the flowering cycle in Pongamia. Recently, Winarto *et al.*^4^, reported four circadian clock genes (ELF4, LCL1, PRR7, AND TOC1) in Pongamia which are key regulators of central oscillator and showed that they were under diurnal regulation. Here, we reported 25 other crucial genes including TFs whose sequence information is not available for Pongamia in the public databases. The clock gene ELF3 is known to form an evening complex (EC) with ELF4 and LUX thus generating circadian rhythms and hence regulate output pathways such as flowering^27^,^28^,^29^,^30^. The peak expression of ELF3 during dawn in leaves of Pongamia is in accordance with the ELF4 expression observed in a previous study^4^. PRR1, PRR3, PRR5, PRR7 and PRR9 are members of PRR gene family and have important roles in the central oscillator^31^. The presence of highly conserved PR and CCT domains in the putative Pongamia PRR proteins implies a similar role to that of *G. soja* and *Arabidopsis* by repressing LCL1 expression in the central oscillator^32^,^33^. LHY and MYB75 are MYB-like transcription factors that play pivotal roles in the morning loop of the central oscillator. These transcription factors belong to the REVEILLE (RVE) family which consists of 11 proteins with conserved MYB-like domain^34^. Since the MYB domain is known for DNA-binding, these transcription factors could play an important role in the DNA-binding activity of Pongamia LCL1. In Pongamia, MYB was actively expressed in all stages of inflorescence development which could be attributed to the anthocyanin metabolism that gives the characteristic colour to the flowers. GI-CO-FT-APT model of signal cascade for floral initiation under long day conditions was well established in *Arabidopsis*^35^. GI protein represses the CYCLING DOF FACTOR 1 (CDFs) thereby allowing the expression of CO protein during late day which eventually activates FT expression^5^. The peak expression of Pongamia GI and FT in the evening as observed in Arabidopsis corroborated the fact that the photoperiod was an ancient and conserved pathway for controlling flowering. Implication of CO-FT module in the control of photoperiodic flowering has also been described in garden pea, sugar beet and woody species such as poplar where this regulatory module has been proposed to mediate other photoperiodic responses such as growth cessation and bud set^36^,^37^,^38^.

Positional cloning and mutation studies on clock genes provided substantial evidence for the role of transcripts showing circadian rhythms in regulating the grain yield, grain weight, number of grains per panicle and flowering time in many cereals^39^,^40^,^41^. Also, in legumes, significant number of transcripts including genes involved in protein, fatty acid synthesis, lipid metabolism and photosynthesis are showing circadian rhythms suggesting the potential roles of circadian clock in flower opening, nectar secretion, seed composition and development^42^,^43^. Pongamia, which is an outcrossing species, through insect - mediated pollination, starts flowering after 3 to 4 years and seed maturation takes about 10 months after flowering. The information provided in this study about circadian clock genes will provide substantial basis for the studies related to modulation/manipulation of flowering time to get shorter vegetative period and prolonged reproductive stage that leads to the extended period of seed production.

Pongamia is believed to contribute to biodiesel production through its ability to biosynthesize and accumulate considerable amounts of unsaturated triacylglycerols (TAGs) in seeds. In this study, the transcripts involved in lipid metabolism were annotated and further analysed to understand oil accumulation and degradation in the seeds of Pongamia which are of great interest for biofuel production. Pongamia takes 9–10 months to form a mature pod after fertilization of the flower. The initial pod and seed development are at low pace with negligible oil content and poor development of the cotyledons. At 175 DAF, the cotyledon development and oil biosynthesis go at constant pace till maturity (300 DAF). Many other Pongamia accessions belonging to different geographical locations had shown similar patterns of oil accumulation during seed development^20^,^44^. During FA biosynthesis, plastidial acetyl CoA and malonyl CoA are converted into long-chain acyl-ACP by a series of reactions involving certain enzymes with ACP as a cofactor. Carboxylation of acetyl-CoA to malonyl-CoA is the first committed step in FA synthesis which is catalysed by a multi-subunit acetyl-CoA carboxylase (ACCase) complex and in turn limits the oil accumulation in the seeds. Our data represented all four subunits of ACCase: alpha carboxyltransferase (CTA), beta carboxyl transferase (CTB) and biotin carboxylase (BC) and also a homomeric isoform. The transcript for homomeric isoform was absent in previous reports on Pongamia, Jatropha and peanut. qPCR analysis showed that these three genes exhibited a coordinated and stable expression pattern throughout the seed development which is in consistent with previous reports on *Arabidopsis*, *B*.*napus and R*.*communis*^45^,^46^. The subsequent formation of plastidial malonyl ACP from malonyl CoA that is catalysed by malonyl-ACP-transferase (MAT) showed maximum expression at stage 2 and stage 3 and decreased thereafter towards the end of the seed development. The activity of ketoacyl-ACP reductase (KAR), which is a component of fatty acyl synthase (FAS) multiprotein complex, is essential for FA biosynthesis and catalyses an NADPH-dependent reduction of 3-ketoacyl-ACP to the 3-hydroxyacyl isomer. Another key enzyme, enoyl-ACP-reductase (EAR) plays a determinant role in establishing the rate of FA biosynthesis^47^. KAR, EAR together with HAD and KAS-II showed a coordinated expression pattern wherein the genes were up regulated at all stages of seed development but showed a downtrend during maturation of the seed (Fig. 6). Similar type of bell shaped expression pattern of FAS genes was also observed in Jatropha seeds^48^. The enzymes SAD, FAD6 and FAD8 biosynthesise oleic acid, linoleic acid and linolenic acid respectively and are crucial for an ideal biofuel feedstock. PpFAD8 is the most abundant transcript represented in our transcriptome data followed by SAD when compared to other lipid biosynthetic enzymes, suggesting the unsaturated FA synthesis potency of Pongamia. However, the expression levels for SAD during seed development were higher than any other enzyme involved in FA synthesis which could be attributed to the low catalytic efficiency of SAD associated with high oleic acid content in Pongamia^45^. Further studies are needed to understand the gene regulation at promoter level and functional characterization of the PpFAD8 protein which provide important clues about oil accumulation patterns in Pongamia seeds. In addition to PpFAD8, other enzymes involved in biosynthesis of unsaturated fatty acids including PpFAD2, PpFAD6 and SAD could be the potential targets for gene engineering to improve oil quality and quantity in Pongamia, where the sequence information can be deduced through our transcriptome data.

The transcripts that encode two acyl-ACP thioesterases that terminate plastid FA synthesis, FATA (responsible for unsaturated FA production) and FATB (for saturated FA production) showed varied expression patterns during the four stages (Fig. 6). The expression of FATA increased significantly from stage 2 to 4 while, FATB decreased after stage 2. This is in agreement with greater plastid production of unsaturated than saturated FAs in Pongamia seeds. However, in Jatropha seeds, FATA expression was at its peak during late developmental stages. Palmitic acid and stearic acid, which are major constituents of cell membrane, also play important role in development of cotyledons which are usually active during 120–210 DAF in case of Pongamia. Our data clearly indicated that the expression of FA biosynthetic genes for saturated FAs followed a typical bell shaped pattern where it increased during stage 1 and was stable during 2^nd^ and 3^rd^ stages which decreased slightly at stage 4 of seed development. Towards the maturity, more unsaturated FAs were synthesized as evidenced from our data on FATB and FATA expression levels and also supported by previous findings on oil content in Pongamia at various seed developmental stages^20^. The free FAs generated by thioesterases in the plastid are esterified to CoA by long-chain acyl-CoA synthetases (LACS) at the plastid envelope. PpLACS showed consistently high level of expression during all the four stages when compared to 150 DAF, suggesting that the oil accumulation was accelerated at 150 DAF. After FA synthesis, a series of membrane-associated reactions assemble the acyl chains into TAG. Glycerol-3-phosphate acyltransferases (GPAT) catalyse sn-1 acylation of glycerol-3-phosphate to yield lysophosphatidic acid (LPA). The second acylation in *de novo* TAG assembly is catalysed by LPA acyltransferase. In Pongamia, the genes involved in TAG assembly including GPAT, LPAT and PAP were mostly expressed during stage 2–4, wherein the maximum oil accumulation has been recorded in our study. However, considerable expression was also noticed during stage I which should account for the membrane lipid biosynthesis during cotyledon development. The final step in TAG biosynthesis is the acylation of diacylglycerol (DAG) to form TAG. Depending on the acyl donor to DAG, two classes of enzymes, diacylglycerol acyltransferases (DGAT) and phospholipid:diacylglycerol acyltransferases (PDAT), can catalyse this crucial step of TAG synthesis^49^. Our results on DGAT and PDAT expression patterns also demonstrate the active involvement of DGAT in Pongamia TAG assembly. Further, the expression patterns of genes involved in β- oxidation revealed that the fatty acid degradation in Pongamia seeds was active during early stages of seed development which could presumptively be responsible for cotyledon development (Fig. 6).

In conclusion, the transcriptome of *Pongamia pinnata* seed along with leaf, pod as well as flower tissues was sequenced and assembled, to maximize the gene representation associated with flowering and lipid biosynthesis. Our data have led to the identification of transcripts, transcription factors involved in various physiological processes and metabolic pathways, which will provide ample information to the database on Pongamia and also aid in the functional and comparative genomic studies to improve oil and seed yield related traits. GI-CO-FT signalling cascade was found to be active in regulating photoperiod control of flowering in Pongamia. The expression patterns of lipid biosynthetic genes at different developmental stages revealed ACCase, SAD and FAD8 as candidate genes during seed maturity and clearly showed that 270–300 DAF was optimum time for seed harvesting. In summary, our results provide an insight into the complex metabolic pathways and regulatory networks involved in different tissues of the Pongamia.

## Methods

### Plant material

*P. pinnata* plantation was established in the experimental farm of Tree Oils India Limited (TOIL), Zaheerabad, Medak district, Andhra Pradesh (latitude 17°36′; longitude 77°31′E; 622 m MSL). High quality, disease free seeds of nearly 600 accessions were collected from various regions of India and planted in the farm. After attaining reproductive phase, the plants which did not give flowering for two years were removed from the farm and remaining plants were assessed for their yield potential for three consecutive years. The highest yielding variety (TOIL 1) was selected as the experimental plant for the current study. Leaves, flowers, pods and seeds at 210 DAF were collected and snap froze in aseptic conditions for transcriptome sequencing.

For gene quantification studies, five year old Pongamia accession (TOIL 1) those were actively flowering were selected. Leaf samples at four time points 00:00, 06:00, 12:00, 18:00 hr of a day and flowers at four developmental phases of infloresence were collected and stored at −80 °C until further use. Seeds of four developmental stages were collected from same accession of Pongamia to quantify lipid biosynthetic genes.

### Transcriptome sequencing

Total RNA was isolated from leaves, flowers, pods and seeds of Pongamia using Agilent plant RNA isolation kit (Agilent Technologies, USA). The concentration, intactness and purity of RNA were checked with Agilent 2100 Bioanalyzer (Agilent Technologies, USA). Samples having RNA integrity number (RIN) value greater than 8 were used for library preparation. Paired-end cDNA library preparation was done according to Illumina TruSeq RNA library protocol outlined in “TruSeq RNA Sample Preparation Guide”. Briefly, 1 μg of total RNA was subjected to Poly A purification of mRNA. Purified mRNA was fragmented for 4 minutes at 94 °C in the presence of divalent cations and reverse transcribed with Superscript III Reverse transcriptase by priming with Random Hexamers (Invitrogen, USA). Second strand cDNA was synthesized in the presence of RNA Polymerase I and RnaseH. The cDNA was cleaned up using Agencount Ampure XP SPRI beads (Beckman Coulter, USA). Illumina adapters were ligated to the cDNA molecules after end repair and the addition of A base. SPRI clean – up was performed after ligation. The library was amplified using 8 cycles of PCR for the enrichment of adapter - ligated fragments. The prepared library was quantified using Nanodrop and validated for quality by running an aliquot on High Sensitivity Bioanalyzer Chip (Agilent Technologies, USA). Sequencing of constructed cDNA library was performed on Illumina NextSeq 500 sequencer. RNA-Seq data were generated in FastQ format.

### Transcriptome assembly, annotation and analysis

Sequencing resulted in the generation of 76 nucleotide raw reads having attached adapter sequences. These raw reads were subjected to filtering through the standard Illumina pipeline. The filtered reads were further subjected for quality control using NGS QC tool kit V 2.3.1 to remove adapters, B-block and low quality bases towards 3′ ends^50^. The high quality filtered reads were *de novo* assembled by Velvet 1.2.10 and Oases 0.2.08 was used for transcript generation^51^,^52^. Genes/proteins were predicted from assembled transcripts using GeneMark software^53^.

MEGA7 was used for the construction of phylogenetic tree using Clustal W and neighbour-joining analysis by taking the known amino acid sequences of all targeted genes and deduced amino acid sequence of Pongamia^54^.

### Pathway analysis and identification of transcription factors

After assembly and clustering, transcript annotation was done by performing BLASTX analysis at an e-value cut-off of 10^−5^ against UniProt-Papilionoideae database^55^. Blast2GO was used to assign GO (Gene Ontology) terms to transcripts on the basis of best significant match with proteins of members of Papilionoideae to impart a broad overview of their functions and categorized into biological process, molecular function and cellular component. Also, KOG (Eukaryotic Orthologous Groups) was used to identify the transcript homologues from other organisms and thus assigning a probable function to transcripts. KAAS (KEGG (Kyoto Encyclopedia of Genes and Genomes) Automatic Annotation Server) was used for metabolic pathway analysis using *Arabidopsis thaliana*, *Arabidopsis lyrata* and *Glycine max* as reference organisms to identify the enriched metabolic pathways in various gene sets^56^. The transcripts were categorized into various transcription factors (TFs) using Transcription factor Family Data Base (TFDB)^57^.

### SSR marker identification

The percentage compositions of the nucleotides A, T, G and C were calculated for each sequence and across the entire distribution of transcripts. Simple Sequence Repeats (SSRs) were detected using MIcroSAtellite tool. SSRs were detected by considering 100 bp flanking sequences on upstream and downstream of SSRs.

### Real-time PCR analysis

Seeds, leaves, roots and flower tissues were collected in triplicates and total RNA was isolated using Spectrum Plant Total RNA isolation kit (Sigma, USA). 1 μg of RNA was used for cDNA synthesis by Revert aid first strand cDNA synthesis kit (Thermo-Fisher Scientific, USA). qRT – PCR was performed on Eppendorf thermal cycler using SYBR FAST qPCR universal master mix (2X) (KAPA Biosystems, USA). Each reaction contained 1 μl of the first-strand cDNA as template in a total volume of 10 μl reaction mixture. List of genes, primer sequences and melting temperatures used in this study were given in Supplementary Table S4 and S5. The amplification program was performed at 95 °C for 30 s followed by 95 °C for 5 s and 55 °C for 30 s (35 cycles). The relative expression was calculated using the formula, 2^−∆∆Ct^, with actin as housekeeping gene for normalisation of data^58^. The fold change values were log transformed with base 2 so that 1.5 fold which corresponds to 0.58 was used to identify differentially expressed genes.

### Quantification of oil

Oil was extracted from four growing stages of Pongamia seeds by soxhlet extraction method using hexane as a solvent as described in Kumar *et al.*^59^. Briefly, seeds (5 g) were ground in a coffee grinder to make powder. Oil was extracted with 150 ml of hexane at distillation temperature for 2 to 3 hours in the Soxhlet extractor using a heating mantle. Hexane was removed from the extracted oil using a rotary evaporator (Heidolph 514-01002-06-0, Germany) at 55 °C under reduced pressure for 30 min.

### Statistics

For qRT PCR analysis, three independent biological replicates with three technical replicates of each were used and the mean ± standard deviation (SD) values were calculated for each sample. The significance of the difference was tested by using Analysis of Variance (ANOVA) and the comparisons were tested with Holm-Sidak method, the level of significance was set to 0.05. Microsoft excel 2013 was used for data processing. Statistical analysis was performed using software, Sigma plat 11.0.

## Additional Information

**Accession Codes**: The data has been submitted to NCBI Sequence Read Archive (SRA) and BioSample databases with BioSample accession number SAMN04212410 and BioProject Id PRJNA299718. The SRA project Id is SRP065225.

**How to cite this article**: Sreeharsha, R. V. *et al.* Unravelling molecular mechanisms from floral initiation to lipid biosynthesis in a promising biofuel tree species, *Pongamia pinnata* using transcriptome analysis. *Sci. Rep.* **6**, 34315; doi: 10.1038/srep34315 (2016).

## Supplementary Material

Supplementary Information

Supplementary Data 1

Supplementary Data 2

Supplementary Data 3

Supplementary Data 4

Supplementary Data 5

## Figures and Tables

**Table 1:** Flowering and circadian rhythms related genes.

Enzyme name	Symbol	U	CDS (bp)	Homology (%)	PP (aa)	Mass (KDa)	pI
Pseudo-response regulator 1	PRR1	1	1710	85 (*G. max*)	269	30.20	5.89
Pseudo-response regulator 5	PRR5	4	2671	82 (*P. trichocarpa*)	687	76.20	6.48
Pseudo-response regulator 7	PRR7	3	2984	83 (*M. truncatula*)	758	83.00	6.27
Phytochrome-interacting factor3	PIF3	3	2005	79 (*G. max*)	288	32.47	8.99
Casein kinase II alpha	CK2A	2	1447	93 (*G. max*)	333	39.30	8.14
Casein kinase II beta	CK2B	9	1597	86 (*M. truncatula*)	284	31.96	5.06
MYB75	MYB75	1	485	88 (*G. max*)	136	15.20	7.80
LHY – MYB related	LHY	3	3305	88 (*P. vulgaris*)	750	82.16	6.07
Chalcone synthase	CS	4	1634	78 (*P. frutescus*)	391	42.96	6.24
Constitutive Photomorphogenic 1	COP1	1	2392	89 (*P. sativum*)	672	75.56	6.94
Clock-associated PAS protein	ZTL	4	2669	91 (*G. max*)	613	66.70	5.44
Phytochrome A	PHYA	1	1966	91 (*G. max*)	332	36.57	6.34
Phytochrome B	PHYB	1	2040	91 (*G. max*)	268	30.75	5.45
GIGANTEA	GI	2	3807	85 (*G. max*)	1159	127.0	6.30
Protein suppressor of PHYA	SPA	1	2267	76 (*A. thaliana*)	529	59.33	5.48
EARLY FLOWERING 3	ELF3	1	1138	81 (*P. sativum*)	316	34.38	8.95
Cryptochrome 1	CRY1	1	1733	88 (*G. max*)	368	41.82	4.96
Cryptochrome 2	CRY2	1	2477	89(*G. max*)	634	72.20	6.16
FLOWERING LOCUS T	FT	1	768	—	178	19.70	5.57
PISTILLATA	PST	1	1089	91 (*G. max*)	226	26.16	9.15
SEPALLATA	SPT	1	1104	86 (M. truncatula)	243	28.10	8.95
APETALA 2	APT2	1	2161	84 (C. cajan)	536	59.54	5.91
APETALA 3	APT	1	1206	90 (G. max)	247	28.4	8.94
CAULIFLOWER/APETALA 1	CAU	1	1132	94 (V. ungiculata)	236	27.40	8.91
FRIGIDA	FRI	1	2000	88 (C. cajan)	549	61.37	6.28
AGAMOUS	AGA	1	971	94 (C. cajan)	243	28.0	9.41

The number of unigenes (U) and the length of the cDNA sequence (CDS) as well as the percentage homology of Pongamia transcripts with other organisms having maximum sequence coverage are represented. The protein parameters like polypeptide (PP) length, mass and pI were deduced using Expasy ProtParam tool.

**Table 2:** Genes involved in lipid biosynthesis.

Enzyme name	Symbol	U	CDS (bp)	Homology (%)	PP (aa)	Mass (KDa)	pI
Acetyl CoA synthesis from pyruvate							
Acetyl CoA synthesis							
PDHC - E2 component	DLAT	7	2350	88 (*M*.*truncatula*)	625	67.72	6.05
PDHC - E1 component α	PDHA	4	1749	82 (*M*.*truncatula*)	429	47.57	6.52
PDHC - E1 component β	PDHB	11	1910	91 (*G*.*max*)	402	43.86	5.75
ATP – Citrate lyase	CL	5	2082	93 (*L. albus*)	615	66.81	7.57
Acetyl CoA synthetase	ACSS	2	2395	88 (*M*.*truncatula*)	754	84.01	5.98
Acetyl CoA acetyltransferase	AAT	2	1612	83 (*P. trichocarpa*)	414	42.86	8.58
**FA synthesis from Acetyl CoA**							
Fatty acid biosynthesis							
ACCase Carboxyl transferase α	ACC-CT	1	2990	85 (*G*.*max*)	727	80.0	6.80
ACCase Carboxyl transferase β	ACC-CT	1	1437	81 (G. max)	278	29.45	8.67
ACCase Biotin carboxylase	ACC-BC	1	2050	95 (*C. cajan*)	540	59.17	6.97
ACCase homomeric protein	ACAC	1	3894	95 (*G. max*)	1297	145.0	6.02
ACP – Malonyl transferase	MAT	1	1614	90 (*A*.*hypogea*)	385	40.0	8.53
Oxoacyl-ACP reductase	KAR	1	1213	79 (*M*.*truncatula*)	260	27.0	7.70
Oxoacyl-ACP synthase II	KAS-II	3	1816	93 (*G*.*max*)	469	49.7	8.31
Oxoacyl-ACP synthase III	KAS-III	1	1840	89 (*G*.*max*)	399	41.0	6.71
Enoyl-[ACP] reductase I	EAR	2	1406	91 (*G*.*max*)	393	40.0	8.64
3-hydroxyacyl-[ACP] dehydratase	HAD	2	932	82 (*M*.*truncatula*)	213	23.0	8.95
Fatty acyl-ACP thioesterase B	FATB	3	1552	91 (*G*.*max*)	417	46.0	6.53
Fatty acyl-ACP thioesterase A	FATA	1	1589	86(*M*.*truncatula*)	350	39.7	5.68
Long-chain acyl-CoA synthetase	ACSL	2	2414	89 (*M*.*sativa*)	662	74.1	6.60
Fatty acid elongation							
Ketoacyl-CoA synthase	KCS	1	2051	85 (*M*.*truncatula*)	521	58.0	9.09
Very-long-chain enoyl-CoA reductase	TER	1	1368	90 (*G*.*max*)	310	36.0	9.63
Palmitoyl-protein thioesterase	PPT	1	1508	85 (*P*.*vulgaris*)	321	36.0	6.46
Very-long-chain hydroxyacyl-CoA dehydratase	PHS1	1	916	88 (*V*.*radiata*)	218	24.7	9.23
Mitochondrial trans-2-enoyl-CoA reductase	MECR	1	1176	90 (*G*.*max*)	318	34.8	7.61
Very-long-chain 3-oxoacyl-CoA reductase	HSDB	2	1178	82 (*M*.*truncatula*)	320	35.8	9.48
Acyl-coenzyme A thioesterase 1/2/4	ACOT	1	1420	85 (*M*.*truncatula*)	429	48.4	8.45
Biosynthesis of unsaturated fatty acids							
Acyl-ACP desaturase	DESA1	1	1245	92 (*P*.*vulgaris*)	385	42.0	5.64
Stearoyl-CoA desaturase (∆9 desaturase)	SAD	3	1400	80 (*M*.*truncatula*)	378	43.5	9.60
ω6 fatty acid desaturase (∆12 desaturase)	FAD2/6	3	1600	88 (*G*.*max*)	363	42.1	8.80
ω3 fatty acid desaturase (∆15 desaturase)	FAD8	5	1433	85 (*G*.*max*)	382	44.5	9.20
**TAG assembly and degradation**							
TAG biosynthesis							
Glycerol-3-phosphate acyltransferase	GPAT	2	1971	91 (*M*.*truncatula*)	376	43.5	9.15
Diacylglycerol O-acyltransferase	DGAT	1	821	90 (*G*.*max*)	155	18.8	9.18
LPA O-acyltransferase	LPAT	1	1293	91 (*M*.*truncatula*)	376	43.5	9.15
Phospholipid: diacylglycerol acyltransferase	PDAT	1	2723	87 (*M*.*truncatula*)	673	75.0	6.28
Phosphatidate phosphatase	PAP	2	3417	77 (*M. truncatula*)	751	83.1	4.86
Fatty acid degradation							
Alcohol dehydrogenase	ADH	1	1680	83 (*M*.*truncatula*)	425	46.2	6.41
Acyl-CoA oxidase	AOX	2	2089	90 (*M*.*truncatula*)	616	69.2	9.01
S- glutathione dehydrogenase	GDH	3	1314	87 (*G*.*max*)	376	40.2	5.42
Aldehyde dehydrogenase	ADH7	1	1930	92 (*G*.*max*)	508	54.1	5.71
Acetyl-CoA acyl transferase	ACAA	2	1731	91 (*G*.*max*)	461	48.5	7.06
3-hydroxyacyl-CoA dehydrogenase	HDH	2	2580	92 (*G*.*max*)	722	78.7	9.11
Acetyl-CoA acetyl transferase	AAT	2	1612	81 (*H. brasiliensis*)	414	42.8	8.58
Acyl-CoA dehydrogenase	ACD	1	2957	76 (*T. cocao*)	466	51.9	8.90
TAG lipase/phospholipase A2	TGL4	2	225	80 (*M*.*truncatula*)	189	20.7	4.49

The number of unigenes (U) and the length of the cDNA sequence (CDS) as well as the percentage homology of Pongamia transcripts with other organisms having maximum sequence coverage are represented. The protein parameters like polypeptide (PP) length, mass and pI were deduced using Expasy ProtParam tool.
