# Supplementary material for: Unravelling molecular mechanisms from floral initiation to lipid biosynthesis in a promising biofuel tree species, Pongamia pinnata using transcriptome analysis
Source: Sci Rep. 2016 Sep 28;6:34315. doi: 10.1038/srep34315 (PMC5039640; doi:10.1038/srep34315)
Supplement: Supplementary Information [file srep34315-s1.pdf]

Unravelling molecular mechanisms from floral initiation to lipid biosynthesis in a promising biofuel tree species, *Pongamia pinnata* using transcriptome analysis

Rachapudi V Sreeharsha[1], Shalini Mudalkar[1], Kambam T Singha, Attipalli R Reddy[*]

Supplementary Fig. S1: (a) Nucleotide composition of Pongamia transcriptome. (b) GC content analysis of Pongamia transcriptome.

a b

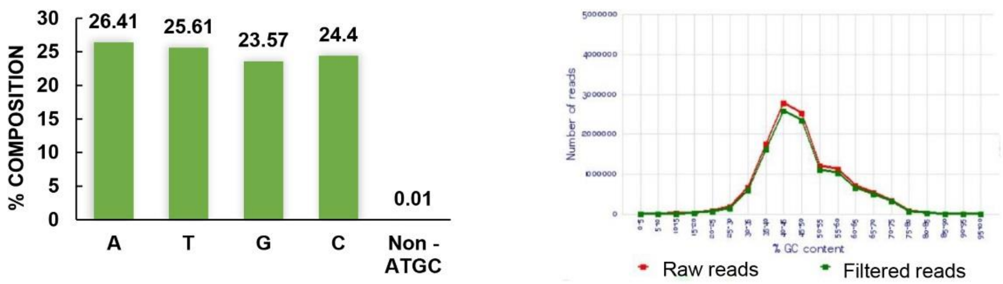

Supplementary Fig. S2: SSR length distribution in Pongamia transcriptome.

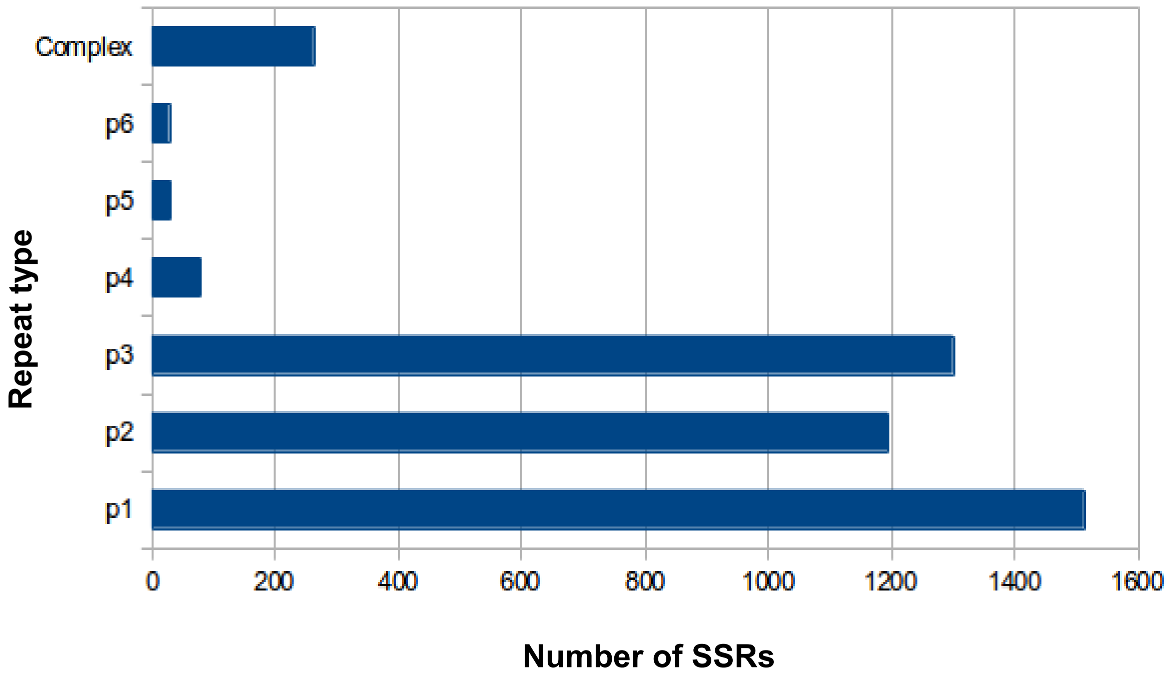

Supplementary Table S3: Transcripts involved in membrane and storage lipid metabolism.

Enzyme name	Symbol	Number of transcripts	Length (bp)
**Fatty acid biosynthesis (10%)**			
Long-chain acyl-CoA synthetase	ACSL	2	2414
Fatty acyl-ACP thioesterase B	FATB	3	1552
Fatty acyl-ACP thioesterase A	FATA	1	1589
Acetyl-CoA carboxylase carboxyl transferase β	accD	1	281
Acyl-[acyl-carrier-protein] desaturase	DESA1	1	1245
Acetyl-CoA carboxylase carboxyl transferase α	accA	1	2990
Acetyl-CoA carboxylase / biotin carboxylase	ACAC	1	4382
fabD	fabD	1	1614
3-oxoacyl-[acyl-carrier-protein] synthase II	fabF	3	1816
3-oxoacyl-[acyl-carrier protein] reductase	fabG	1	1213
3-oxoacyl-[acyl-carrier-protein] synthase III	fabH	1	1840
Enoyl-[acyl-carrier protein] reductase I	fabI	2	1406
3-hydroxyacyl-[acyl-carrier-protein] dehydratase	fabZ	2	932
**Fatty acid elongation (4%)**			
3-ketoacyl-CoA synthase	KCS	1	2051
Very-long-chain enoyl-CoA reductase	TER	1	1368
Palmitoyl-protein thioesterase	PPT	1	1508
Very-long-chain (3R)-3-hydroxyacyl-CoA dehydratase	PHS1	1	916
Mitochondrial trans-2-enoyl-CoA reductase	MECR	1	1176
Very-long-chain 3-oxoacyl-CoA reductase	HSD17B12	2	1178
Acyl-coenzyme A thioesterase 1/2/4	ACOT	1	1420
**Biosynthesis of unsaturated fatty acids (5%)**			
Omega-6 fatty acid desaturase (delta-12 desaturase)	FAD6	1	1533
Omega-6 fatty acid desaturase (delta-12 desaturase)	FAD2	2	1600
Stearoyl-coa desaturase (delta-9 desaturase)	SCD	1	1400
Omega-3 fatty acid desaturase (delta-15 desaturase)	FAD8	5	1433
**Arachidonic acid metabolism (4%)**			
Glutathione peroxidase	E1.11.1.9	2	794
γ - glutamyltranspeptidase / leukotriene-C4 hydrolase	GGT1_5	2	594
Microsomal prostaglandin-E synthase 2	PTGES2	1	642
Cytosolic prostaglandin-E synthase	PTGES3	1	903
Leukotriene-A4 hydrolase	LTA4H	1	1560
**Linoleic acid metabolism (2%)**			
Lipoxygenase	LOX2S	2	2676
Linoleate 9S-lipoxygenase	LOX1_5	2	3134
**α - Linolenic acid metabolism (5%)**			
OPC-8:0 CoA ligase 1	OPCL1	1	1912
Hydroperoxide dehydratase	AOS	2	1732
Allene oxide cyclase	AOC	2	1187
Jasmonate O-methyltransferase	E2.1.1.141	2	1343
12-oxophytodienoic acid reductase	OPR	1	1649
Alpha-dioxygenase	DOX1	1	2005
Hydroperoxide lyase	HPL	1	1604
**Glycerolipid metabolism (15%)**			
Phosphatidate phosphatase LPIN	LPIN	2	3417
Dihydroxyacetone kinase	E2.7.1.29	1	966
Diacylglycerol kinase (ATP)	dgkA	2	2596

Aldehyde dehydrogenase (NAD+)	E1.2.1.3	2	1755
Glycerol-3-phosphate acyltransferase	GPAT	2	1971
Diacylglycerol O-acyltransferase 1	DGAT1	1	821
Digalactosyldiacylglycerol synthase	E2.4.1.241	2	1038
Alpha-galactosidase	E3.2.1.22B	4	1728
Glycerol-3-phosphate O-acyltransferase 3/4	GPAT3_4	1	1293
Aldehyde reductase	E1.1.1.21	1	1423
Glycerol kinase	glpK	1	1873
D-glycerate 3-kinase	GLYK	1	1678
1-acyl-sn-glycerol-3-phosphate acyltransferase	plsC	1	1173
Phospholipid:diacylglycerol acyltransferase	E2.3.1.158	1	2723
Lysocardiolipin and lysophospholipid acyltransferase	LCLAT1	1	773
1,2-diacylglycerol 3-beta-galactosyltransferase	E2.4.1.46	2	1663
UDP-sulfoquinovose synthase	SQD1	2	1840
Sulfoquinovosyltransferase	SQD2	1	2110
2-acylglycerol O-acyltransferase 2	MOGAT2	1	1304
Glycerol-3-phosphate O-acyltransferase	ATS1	2	1851
**Glycero phospholipid metabolism (14%)**			
Lysophospholipase II	LYPLA2	2	1085
Glycerol-3-phosphate dehydrogenase	glpA	1	2307
Glycerol-3-phosphate dehydrogenase (NAD+)	GPD1	2	2142
Monolysocardiolipin acyltransferase	TAZ	2	1468
Lysophosphatidic acid acyltransferase	AGPAT	2	1188
Choline/ethanolamine kinase	CHK	2	1445
Phosphoethanolamine N-methyltransferase	E2.1.1.103	1	1722
Phosphatidate cytidylyltransferase	E2.7.7.41	1	1921
Ethanolamine kinase	ETNK	1	1580
Ethanolamine-phosphate cytidylyltransferase	PCYT2	2	1868
Choline-phosphate cytidylyltransferase	PCYT1	2	1326
CDP-diacylglycerol--glycerol-3-phosphate 3-phosphatidyltransferase	pgsA	1	1243
Phosphatidylserine decarboxylase	psd;	1	782
1-acyl-sn-glycerol-3-phosphate acyltransferase	plsC;		1173
Lysophospholipase III	LYPLA3	1	1419
Mn-dependent ADP-ribose/CDP-alcohol diphosphatase	ADPRM	1	371
Phosphatidylserine synthase 2	PTDSS2	1	1989
Methylene-fatty-acyl-phospholipid synthase	OPI3	1	618
Cardiolipin synthase	CRLS	2	1102
Ethanolaminephosphotransferase	EPT1	1	1455
CDP-diacylglycerol--inositol 3-phosphatidyltransferase	CDIPT	1	1007
Phosphatidylglycerophosphatase GEP4	GEP4	1	1708
**Fatty acid degradation (8%)**			
Alcohol dehydrogenase	AD	1	1680
Acyl-CoA oxidase	ACO	2	2089
S-(hydroxymethyl)glutathione dehydrogenase	frmA	3	1314
Aldehyde dehydrogenase family 7 member A1	ALDH7A1	1	1930
Acetyl-CoA acyltransferase 1	ACAA1	2	1731
3-hydroxyacyl-CoA dehydrogenase	MFP2	2	2580
Acetyl-CoA C-acetyl transferase	--	2	1612
Alcohol dehydrogenase class-P	ADH1	2	1408
Acyl-CoA dehydrogenase	ACADM	1	2957
**Ketone bodies synthesis and degradation (2%)**			
Hydroxymethylglutaryl-CoA synthase	E2.3.3.10	2	1570
Hydroxymethylglutaryl-CoA lyase	E4.1.3.4	2	1690
**Cutin, suberine and wax biosynthesis (9%)**			
Peroxygenase	PXG	2	975

Omega-hydroxypalmitate O-feruloyl transferase	HHT1	1	1564
Fatty acid omega-hydroxylase	CYP94A1	1	1672
Aldehyde decarbonylase	K15404	7	1846
Fatty acid omega-hydroxy dehydrogenase	ACE	2	1885
Fatty acyl-CoA reductase	FAR	3	1802
Fatty acid omega-hydroxylase	CYP86A4S	3	2070
**Steroid biosynthesis (9%)**			
Sterol 14-demethylase	CYP51	1	2035
Squalene monooxygenase	SQLE	2	1802
4,4-dimethyl-9β, 19-cyclopropylsterol-4α-methyl oxidase	SMO1	1	1408
Delta24-sterol reductase	DHCR24	1	2001
Sterol-4 alpha-carboxylate 3-dehydrogenase	E1.1.1.170	2	581
Cycloartenol synthase	E5.4.99.8	1	2642
7-dehydrocholesterol reductase	DHCR7	1	1773
Cycloeucalenol cycloisomerase	CPI1	1	1262
Sterol 24-C-methyltransferase	E2.1.1.41	1	1318
4-alpha-methyl-delta7-sterol-4alpha-methyl oxidase	SMO2	1	994
Lathosterol oxidase	SC5DL	1	1251
Lysosomal acid lipase/cholesteryl ester hydrolase	LIPA	1	1415
Cholestenol delta-isomerase	EBP	1	895
24-methylenesterol C-methyltransferase	E2.1.1.143	2	1377
Farnesyl-diphosphate farnesyltransferase	FDFT1	1	1679
**Ether lipid metabolism (4%)**			
Lyso-PAF acetyltransferase	LPCAT1_2		938
Lysophospholipid acyltransferase	LPT1	2	1992
Phospholipase C	plcC		1467
TAG lipase / steryl ester hydrolase / phospholipase A2	TGL4	2	225
Phospholipase D1/2	PLD1_2	2	553
Secretory phospholipase A2	PLA2G	1	497
Ethanolamine phosphotransferase	EPT1		1455
**Sphingolipid metabolism (10%)**			
Sphinganine C4-monooxygenase	SUR2	2	1435
Ceramide synthetase	CERS	3	1312
Dihydroceramidase	ACER3	1	1406
Beta-galactosidase	lacZ	1	3324
Sphingolipid delta-4 desaturase	DEGS	1	1240
Ceramide kinase	E2.7.1.138	1	2206
Sphingosine-1-phosphate phosphatase 1	SGPP1	2	1536
Serine palmitoyltransferase	SPT	3	1793
Neutral ceramidase	ASAH2	1	2752
Non-lysosomal glucosylceramidase	GBA2	2	3425
Sphinganine-1-phosphate aldolase	SGPL1	1	204
Sphingosine kinase	SPHK	1	1041
Beta-galactosidase	GLB1	1	1169
3-dehydrosphinganine reductase	E1.1.1.102	1	1274

Supplementary Table S4: Primer sequences used for qRT PCR studies for quantification of lipid biosynthetic genes during different developmental stages of the Pongamia seed.

Gene	Forward primer	Reverse primer	Tm	Product length
**ACC-CTA**	TCCTTTCCCCCTCTTGTTCT	CCAAGGTTCTCAAAGGGACA	56	189
**ACC-CTB**	ATCACCCGTTTGGTCGAATA	GCTGTCACCCCACCTGTAGT	55	178
**LACS**	CTTGGAAAGTTTGGGTTGGA	TCCCAGCATTTGCTTAATCC	55	180
**MAT**	TTCTCTCTCCGCCATGTCTT	GAAACGAAGTGGAGGCTTTG	56	190
**KAR**	ATCCCTCAGATCCGTCATTG	GGCTCCGGTAACTACCACAA	56	176
**HAD**	TGGCCTTTCAGGAAAATGTC	ATTCCCAACCGTCATGGATA	55	180
**FAD2**	TAACAACCCACCAGGGAGAG	ATTGCAAGAATCCCAGCATC	54	189
**FAD8**	CTGGGCTCTCTTTGTTCTGG	GGTTTTGATGGTGGGTTTTG	56	190
**FATA**	CGAGAGTGCCAACAAGATGA	CCCGGTTTATCTCAAGTCCA	55	180
**FATB**	ACATTGGCTGGATTCTGGAG	TCAGCACCATCTTCAAGTCG	56	170
**SAD**	GGTGGCTGTGGCACTTTATT	CAACTGGATTCCCCTGAAGA	56	180
**TGL4**	AGCTTCCGAAAATGGTGATG	GCACCAAATTCGAGGAAAAA	55	189
**KASIII**	ACTGTTTCCCCTTCCCAATC	GCCTGAAAGAACTCGTCGTC	56	183
**KASII**	TGAGGTAGCACAAGCAGTGG	CTGGTGTTCTGGTTGGGTCT	55	200
**EAR**	TGGTCCACTGAGAAGTCGTG	TCCAACACCCATAGCATTCA	56	205
**ECH**	ATCCTGTGCAGTTTCCAACC	TTGTTGGAGCCCATTTCTTC	56	153
**ACD**	GGGCTTCAATTTATGCAGGA	AGCCTCGTGCATTAGCATCT	56	172
**HDH**	ACCAAGAGGTCGTGGAGATG	ATCTGCCCAGAAAACAATGC	57	158
**GPAT**	GTGGGACTGGTTTTGCTGAT	AAGAAGGCTACGGGATTCGT	57	193
**LPAT**	TCCACAACACCACAAAGGAA	ATCCGCCATTCAACAAGAAC	56	181
**PAP**	CAAGGAGGTGGAGGATTCAA	CGAACAAAACAACCCCAACT	56	202
**DGAT**	ACAGCAACAGCCTTCGGTAT	TTCGATTTGGTGATCGTGAA	57	154
**PDAT**	TCAGGCCTGTCTCTGGACTT	CTTAGCGCTTGATCCCTCAC	57	175

Supplementary Table S5: Primer sequences used for qRT PCR studies for quantification of spatial and temporal expression of flowering related genes of Pongamia.

Gene	Forward primer	Reverse primer	Tm	Product length
**PRR1**	CTCGTGTGAGGGGACAATTT	GCCCTTCATCCTCACAACAT	55	179
**PPR5**	TGATTCAGTTGGCATGGTGT	TCGCTTGCAGAATTGTCATC	55	192
**PPR7**	TGTCTAGTGGGGAGCAGCTT	ACAGCATCCATCCCTTGAAC	55	185
**PIF3**	CCAATTCCTCATGCACCTTT	GAGCTCGTGGGAACTGAGAC	55	165
**CK2A**	ATCCTTCACAAACGCCAGAT	CCCTTCCCACTTTTCGTACA	55	169
**CK2B**	CCCACCTGTTCCTGATGACT	AGCAGCGTGCCATAGAGAGT	55	184
**MYB75**	GCAAGATGCGGAATACACAA	CTGATGAAGCCAAACCCACT	55	156
**LHY**	CGTGTCACCAAAAATGAACG	CGGAACCCAACTCACCTAAA	55	184
**CS**	TTGTTCTCAGCGAGGTCCTT	GGGTCAACCCAAGTCAAAGA	55	192
**COP1**	AAGGCCACTGCTGTTCTTGT	CGCAAGCAAAAAGTTGATGA	55	198
**ZTL**	GGGTTCTACAGCACCTCCAA	AGAATGGTTTGCCAGAATGC	55	159
**PHYA**	TGGACGGATTTCCCTAACTG	TGTTGCAGAGCACACAATGA	55	162
**PHYB**	CATTCCCATTTGGCAGAACT	GACTTCCCCATAGCCTCCTC	55	202
**G1**	AAAAAGCCAAGAGGTGCAGA	TCACAGGCAAGAGCACAAAC	55	167
**SPA**	AAAAAGGCCAGGAACAACCT	CCCGATCAAAGCTCAGAGAG	55	162
**ELF3**	GCCAGCTATTTTGCTCGAAG	CCTCAACTGCATTCTCAGCA	55	179
**CRY1**	ATCATCCATGGAATGCACCT	CTCTCGAAGCTGCCTCTTGT	55	153
**CRY2**	TCAGCTGTGGAGCACATTTC	GGACCCTCCAAAAGTTGTCA	55	219
**FT**	AGGATGGGCACATAGTCGTC	CGGACTCCATCGTTACGTTT	55	195
**PST**	CAAGGTTGTTCGTGAGCGTA	CATTAGGATGGCTTGGCTGT	55	236
**SPT**	GAACGCCATCTTGGAGACAT	TTCTGCCGGGTAATCCATAG	55	165
**APT**	AAACCTGCCAAAGAGCTTGA	TTGTGGACCTCACTTGCTTG	55	181
**CAU**	TCACAGGGAAATTGGACCAT	CTGCGGGTACGAATTTGTTT	55	170
**FRI**	CCAAGAGGTGCAGAAGCTAG	GCAAGAGCACAATTGTCAGC	55	189
**AGA**	GCCAAAGAGCTTGACAAGGC	GTGGACTTGGCTTGGCTGTG	56	194
